# High-Resolution 3D Fabrication of Glass Fiber-Reinforced Polymer Nanocomposite (FRPN) Objects by Two-Photon Direct Laser Writing

**DOI:** 10.1021/acsami.1c21708

**Published:** 2022-04-08

**Authors:** Tiziana Ritacco, Wera Di Cianni, Dario Perziano, Pietro Magarò, Annalisa Convertino, Carmine Maletta, Antonio De Luca, Alberto Sanz de León, Michele Giocondo

**Affiliations:** † Institute of Nanotechnology—Nanotec Consiglio Nazionale delle Ricerche—Sede di Cosenza. Ponte P. Bucci - Cubo 33C, Rende 87036, Italy; ‡ Physics Department, University of Calabria, 87036 Arcavacata di Rende, CS, Italy; § Institute for Microelectronics and Microsystems—IMM Consiglio Nazionale delle Ricerche, via del Fosso del Cavaliere 100, 00133 Roma, Italy; ∥ Department of Mechanical, Energy and Management Engineering, University of Calabria, Cubo 44C, Arcavacata di Rende 87036, Italy; ⊥ Departamento de Ciencia de los Materiales, I. M. y Q. I., IMEYMAT, Facultad de Ciencias, Universidad de Cádiz, Campus Río San Pedro, s/n, 11510 Puerto Real, Cádiz, Spain

**Keywords:** direct laser writing, two-photon absorption, nanocomposites, nanofabrication, silica nanowires, nanomechanics

## Abstract

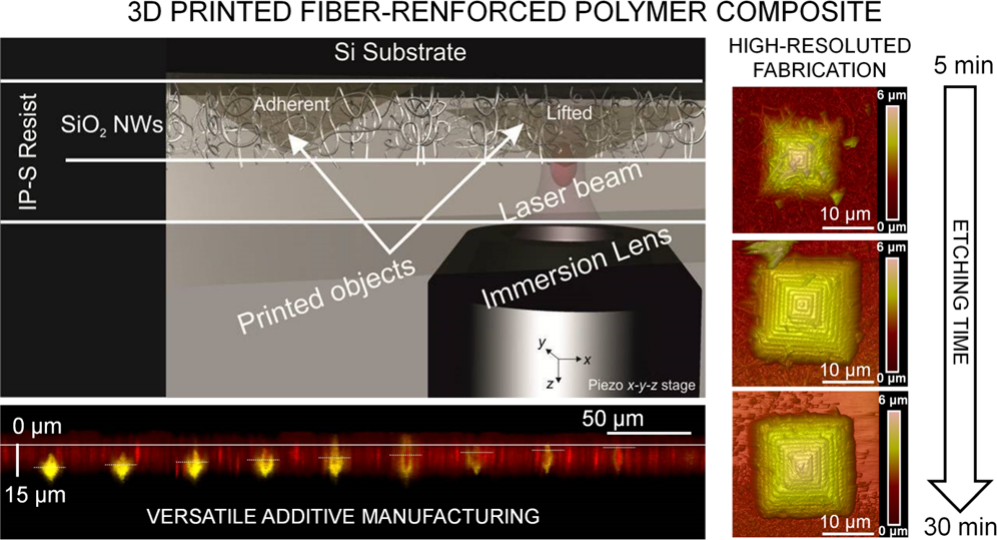

This paper reports on the nanofabrication of a fiber-reinforced polymer nanocomposite (FRPN) by two-photon direct laser writing (TP-DLW) using silica nanowires (SiO_2_ NWs) as nanofillers, since they feature a refractive index very close to that of the photoresist used as a polymeric matrix. This allows for the best resolution offered by the TP-DLW technique, even with high loads of SiO_2_ NWs, up to 70 wt %. The FRPN presented an increase of approximately 4 times in Young’s modulus (8.23 GPa) and nanohardness (120 MPa) when compared to those of the bare photoresist, indicating how the proposed technique is well-suited for applications with higher structural requirements. Moreover, three different printing configurations can be implemented thanks to the use of silicon chips, on which the SiO_2_ NWs are grown, as fabrication substrates. First, they can be effectively used as an adhesive layer when the laser beam is focused at the interface with the silicon substrate. Second, they can be used as a sacrificial layer, when the laser beam is focused in a plane inside the SiO_2_ NW layer. Third, only the outer shell of the object is printed so that the SiO_2_ NW tangle acts as the internal skeleton for the structure being fabricated in the so-called shell and scaffold printing strategy.

## 1 Introduction

The two-photon direct laser writing (TP-DLW) technique has revolutionized the three-dimensional (3D) micro/nanofabrication of polymeric materials, allowing the 3D printing of structures with a resolution below the diffraction limit (down to 50 nm). A femtosecond pulsed infrared laser is focused on a UV photosensitive prepolymer (resist) and triggers the polymerization by two-photon absorption (TPA).^1−13^ TPA is a nonlinear, third-order optical absorption phenomenon theorized in the early ‘30s of the past century by Göppert Mayer.^11^ The smallest printable volume (volume pixel or voxel) defines the achievable high spatial resolution, which is due to the square dependence of the radiation intensity that further narrows the Gaussian laser beam intensity profile. Therefore, only in the inner volume of the focus figure, where the intensity is the highest, it is possible to promote the electronic transition and the material polymerization.^14−16^

To confer and tune the mechanical, electric, and optical properties of the created structures, scientists have been exploring the possibility to embed nanofillers in the form of fibrils or particles into different polymeric matrixes, allowing us to fabricate structures suitable for integrating in medical and microfluidic devices, electronic components, and even small satellites.^17−32^

In the case of transparent nanofillers, as silica nanowires (SiO_2_ NWs), they could be embedded in polymeric matrices and processed by TP-DLW, allowing for the best performance in terms of resolution, when their refractive indexes are perfectly matched, eliminating the light scattering effects. This optimal condition could be achieved by tuning the refractive index of the polymer during the synthesis^21,22^ or that of the SiO_2_ NWs by adding a suitable dopant in the production process.^23−25^

In this work, to be considered as a proof of concept, we used a commercially available photoresist suitable for TP-DLW (IP-S from NanoScribe) featuring a refractive index of 1.499, as reported by Gissibl et al.,^24^ practically coincident with the refractive index of the bare SiO_2_ NWs and the fused silica coverslip commonly used as fabrication substrates. To prepare our samples, we used TP-DLW on silicon substrates with silica nanowires grown on the top and soaked with the photoresist. The refractive index matching between the polymer and the nanofiller allows us to exploit the capabilities of the TP-DLW technique, with a resolution considerably below the diffraction limit, in the order of hundreds of nanometers. This enables the possibility of printing actual 3D trajectories and manufacturing complex, high-resolution 3D objects actually made of a fiber-reinforced nanocomposite. Other authors have demonstrated the possibility to include nanowires directly in the photopolymerizable resin, to confer peculiar properties to the TP-DLW-fabricated structures.^25−30^ However, in general, a minimal percentage of nanofillers can be embedded in the presence of a refractive index mismatch, as the light scattering would jeopardize the TP-DLW resolution. Our experiments demonstrate that our approach consents to realize 3D structures with reinforced structural properties and sub-micrometric resolution. Topographical and mechanical characterization allowed evaluating the maximum resolution of these nanocomposites in 3D fabrication, comparable to that of the bare IP-S resin, and their hardness and elastic properties, obtaining a notable increase in the mechanical properties. The development of such a fiber-reinforced polymer nanocomposite (FRPN) for TP-DLW is expected to have direct applications in different fields such as microfluidic devices,^31^ microelectromechanical systems,^32^ or mechanical metamaterials.^33,34^ In these cases, expanding the library of the materials processable by TP-DLW will allow fabricating higher performance devices.

## 2 Experimental Section

### 2.1 Materials

Poly(propylene glycol methyl ether acetate) (PGMEA), isopropanol (IPA), 49% hydrofluoric acid (HF) solution, ammonium fluoride (NH_4_F), silane (SiH_4_), and hydrochloric acid (HCl) were purchased from Sigma Aldrich. Edible red dye E120 is available at grocery stores. High-purity distilled water was used to prepare all the solutions.

### 2.2 Synthesis and characterization

Silica nanowires (SiO_2_ NWs) on silicon (Si) wafers were produced by thermal annealing of Si NWs grown by plasma-enhanced chemical vapor deposition (PECVD). First, to induce the Si NW growth, a 2 nm-thick Au film was deposited by physical vapor deposition (PVD) onto the Si substrate prior to growth. The growth was performed using pure SiH_4_ as a precursor at a total pressure of 1 Torr and substrate temperature of 350 °C. A 13.56 MHz radiofrequency device with a power density of 50 mW cm^–2^ was used to create the plasma. Under these growth conditions, tens of μm-long Si NWs with an average diameter of 30–300 nm at the bottom and tapered shape are obtained.^21^ After the growth, the Si NWs were thermally oxidized in a convection oven (controlled O_2_ atmosphere) at 980 °C for 8 h to form SiO_2_ NWs.^22,30^

**Figure 1:**
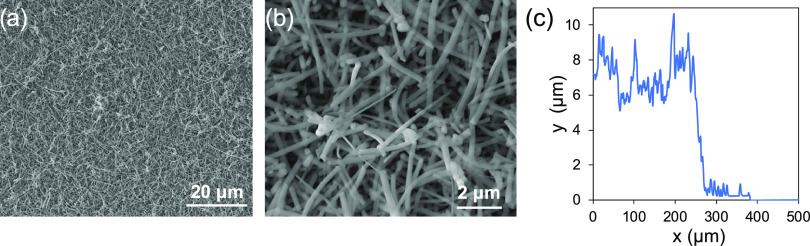
(a, b) SEM images of SiO_2_ NWs grown on the Si substrate; in (b), it is possible to distinguish the NWs with a homogenous distribution of diameters in the range of 30–300 nm, and (c) profilometer measure of the thickness of the SiO_2_ NW layer on the Si substrate. The total thickness is about 8 μm.

The morphological characterization of the SiO_2_ NWs was performed using an FEI Quanta FEG 400 F7 scanning electron microscope (SEM). The average thickness of the NW layer over the fabrication substrate, measured using a profilometer (Veeco DEKTAK 8, Advanced Development profiler), is about 8 μm (Figure 1).

### 2.3 Fabrication Procedure via TP-DLW

TP-DLW was performed with a Photonic Professional GT system, from Nanoscribe GmbH, based on an inverted microscope, using a 150 mW pulsed erbium laser at 780 nm (pulse duration 100 fs, 80 MHz repetition rate).

The used photoresist is the commercial IP-S from Nanoscribe GmbH. It is composed by 95% of carbamate and methacrylate monomers, less than 5% γ-butyrolactone, and less than 1% of photoinitiator.^35^ Among all the photoresists available in our lab, IP-S better matches the SiO_2_ NW refractive index,^18^ evaluated by the optical contrast between the resist and the filler in optical microscopy images.

**Figure 2:**
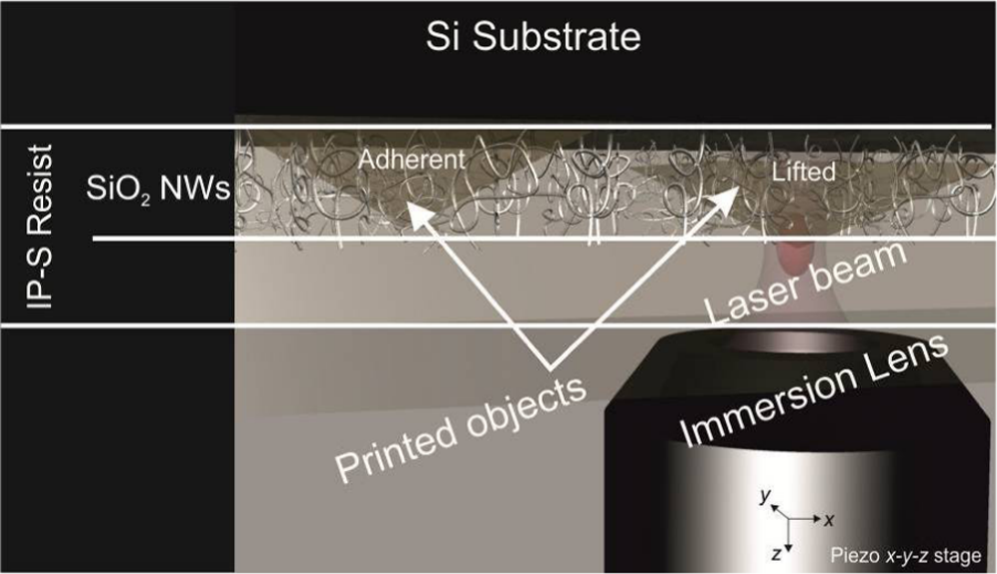
Scheme of the printing configuration: the SiO_2_ NW layer grown on the silicon chip is soaked with a drop of IP-S resist, acting as an immersion fluid for the front lens. The object is fabricated upside down, starting at the Si/SiO_2_ NW interface or a few microns above it, depending on whether a permanently adherent or a removable structure is required.

**Figure 3:**
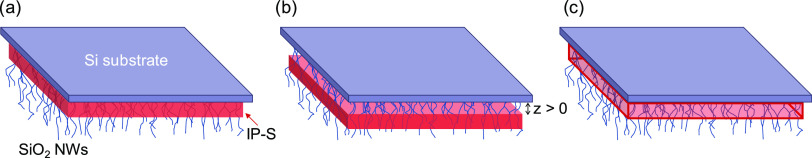
Objects were printed via TP-DLW with three different approaches: (a) first layer is printed at the interface with the Si for adhered structures to the substrate; (b) first layer is printed leaving a gap with the the Si substrate (*z* > 0); in this case, the object can be detached after the etching of the SiO_2_ between the resin and the Si substrate, which acts as a sacrificial layer; (c) same case as (a) but printing only the outer shell of the structure; in this case, the SiO_2_ NW tangle acts as an internal scaffold (shell and scaffold approach) for the outer shell, preventing its collapse. The unpolymerized material remaining inside the shell is cured afterward by single-photon absorption using a UV source.

The SiO_2_ NW layer on the silicon chip is soaked with a drop of IP-S and put on the inverted microscope stage. A 25× and a 63× objectives with a numerical aperture (N.A.) of 1.2 and 1.4, respectively (Zeiss, Plan Apochromat), in the immersion mode, were used, according to the scheme in Figure 2. In this configuration, the resist acts as an immersion fluid for the front lens and the object is fabricated upside down. Various simple parallelepipeds (base 500 × 500 μm^2^ and heights of 2, 8, and 12 μm) and a matrix of pyramids (20 × 20 × 6 μm^3^) were designed and printed in different *z-*positions, namely (a) adherent at the interface with the Si wafer and (b) at a few microns above the substrate, anchored to the SiO_2_ NWs only. Both samples were printed as fully solid objects (Figure 3a,b). The parallelepiped was also printed as a simple shell, in a “shell and scaffold” approach (Figure 3c).

It is important to point out that taller FRPN structures can be made using thicker SiO_2_ layers or by mixing in the resist the SiO_2_ NWs removed from the growth substrate. In the last case, the in-dip configuration, where the resist acts as an immersion fluid for the focusing lens, could be implemented provided that a more accurate index matching is achieved, considering the typical working distance of the focusing lens in the order of a few hundreds of micrometers. This would lead to the possibility to fabricate 3D PFRN structures whose *z* size is limited only by the *z* range of the sample holder stage (several millimeters). In our case, structures shorter than 8 μm are fully made using the FRPN, while the higher structures have an FRPN base and a fully cured resist made of bare cured resist, in the rest of the volume.

After the laser exposure, the samples are developed in PGMEA for 10 min to remove the exceeding unpolymerized resist and then washed in IPA during 3 min to completely remove the PGMEA from the polymer.

### 2.4 Postprocessing of the FRPN with HF Buffer

The polymerized and developed FRPN still contains an excess of SiO_2_ NWs, which are eliminated in a HF buffer. For this purpose, samples were immersed in an etching solution (85 mL of distilled H_2_O, 10 mL of HCl, and 5 mL of buffered HF solution) for times ranging from 15 to 30 min, controlling the etching progress of the SiO_2_ NWs at regular times. The buffered HF solution was prepared using 40 g of NH_4_F, 60 mL of distilled H2O, and 10 mL of 49% HF solution.

### 2.5 Topographic Characterization

The topography of the printed samples was analyzed by profilometry (Veeco DEKTAK 8, Advanced Development profiler), atomic force microscopy (AFM, Bruker Bioscope Catalyst), scanning confocal microscopy (Zeiss LSM 710), and SEM (FEI Quanta FEG 400 F7). AFM measurements were carried out operating in the noncontact mode under ambient conditions. An 8 nm-radius silicon tip (model: RTESP-300, *k* = 40 N m^–1^, resonant frequency *f*_0_ = 300 kHz), on an antimony (n)-doped silicon cantilever, was employed to scan the sample with a rate of 0.15 Hz.

To reveal the polymerized 3D structures inside the NW network by confocal microscopy, a droplet of water solution containing an edible red dye (E120) was casted on the NWs. The fluorescence of both the polymerized IP-S and the dye, presenting a peak at λ_P_ = 540 nm and λ_D_ = 630 nm, respectively, was excited using a 514 nm laser. A 63× oil-immersion N.A. 1.4 objective was used for the imaging.

### 2.6 Mechanical Characterization

The mechanical characterization was carried out on the nanoscale by the instrumented nanoindentation method on a (500 × 500 × 8 μm^3^) parallelepiped, to perform the measurement on the thickest nanocomposite sample, with the aim of measuring the nanohardness and elastic modulus of the fiber-reinforced polymer nanocomposite compared to the bare resist. The tests were performed using a nanoindentation platform (NHT, Anton Paar) equipped with a sphero-conical tip, with a radius of 20 μm. A spherical tip was adopted instead of more common sharp tips (such as Berkovich or Vickers) to limit the maximum penetration depth due to the reduced thickness of the nanocomposite samples. The maximum indentation load (*P*_max_) was varied between 0.5 and 10 mN with a holding time of 10 s. Results are presented as the average of multiple indentations together with the corresponding scatter band. More details about the mechanical characterization are in the Supporting Information.

## 3 Results and Discussion

### 3.1 Nanofabrication of FRPN via TP-DLW

**Figure 4:**
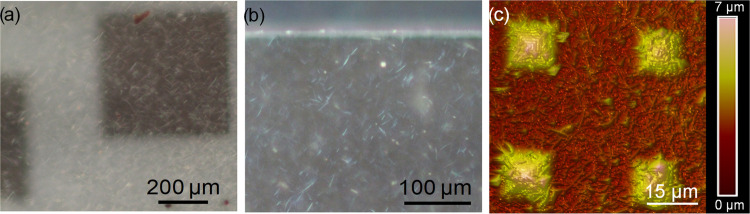
(a, b) Optical microscopy images of the 500 × 500 × 2 μm^3^ parallelepiped and (c) AFM image of the 20 × 20 × 6 μm^3^ pyramids printed through the SiO_2_ NW layer. In (b) and (c), the SiO_2_ NWs embedded in the polymeric matrix can be observed.

A parallelepiped and a matrix of pyramids were chosen as sample structures. The first one demonstrates the effectiveness of the SiO_2_ NWs as a structural scaffold when only the outer shell is printed, while the second one demonstrates the maximum achievable resolution by TP-DLW. These structures, imaged by optical microscopy and AFM, are presented in Figure 4.

**Figure 5:**
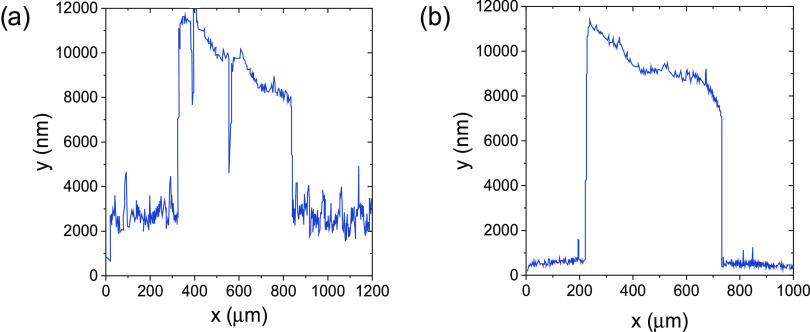
Profilometer measurements of an adhered (500 × 500 × 12 μm^3^) parallelepiped at the SiO_2_ NW interface (a) before and (b) after 15 min of immersion in HF buffer. A decrease in the roughness of the baseline evidences the successful etching of the SiO_2_ NWs.

In the case of objects built directly adhered to the Si substrate (Figure 3a), a subsequent 15 min bath in HF buffer completely etches the exposed SiO_2_ NWs, whereas the polymeric matrix adhered to the Si substrate protects those embedded. It was checked that the objects are firmly held by the SiO_2_ NW layer even after prolonging the immersion times in the etching solution well beyond the optimum, estimated in about 15 min. The effect of the HF buffer on the SiO_2_ NWs was studied by profilometry (Figure 5). In this case, a structure of 12 μm height was printed.

**Figure 6:**
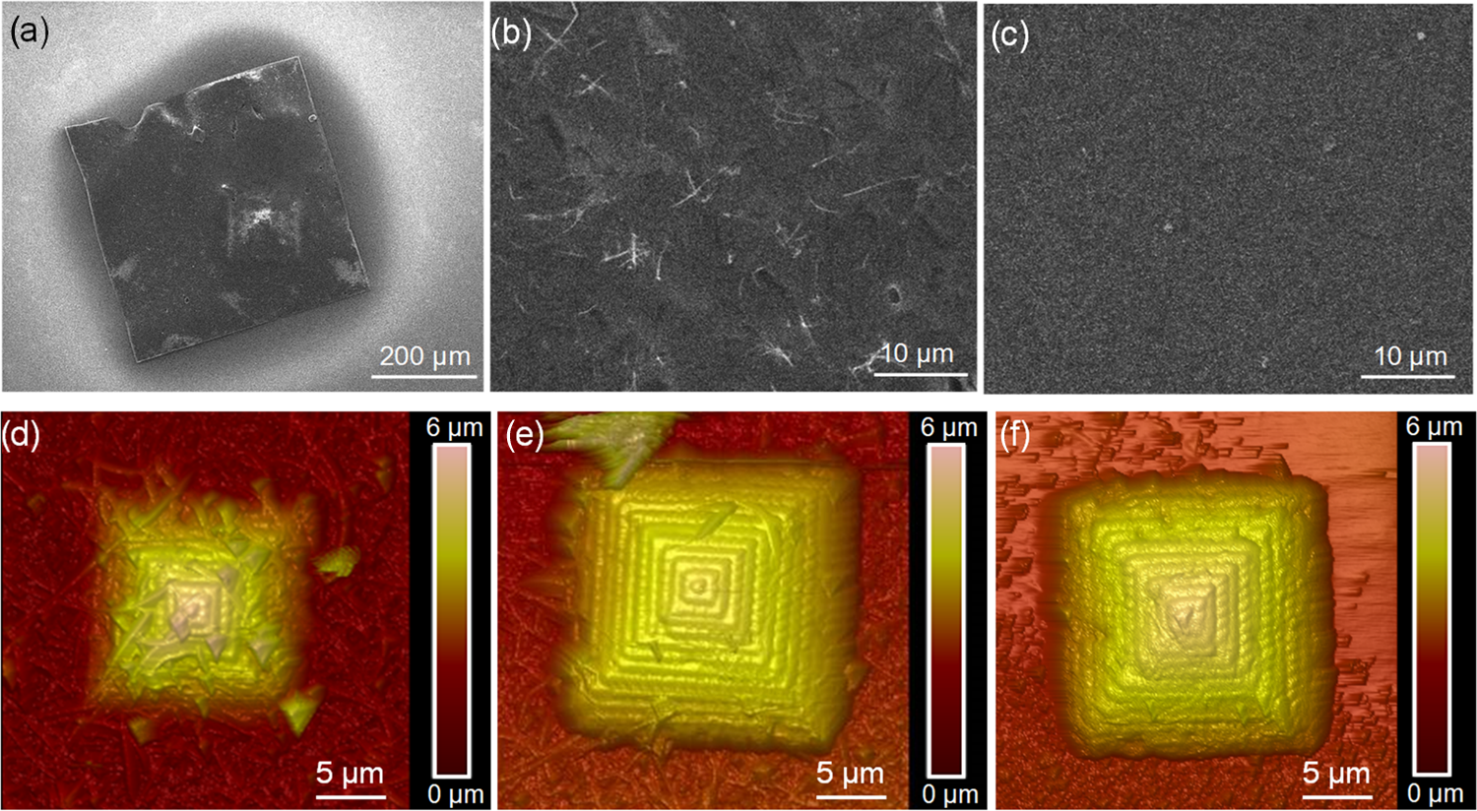
SEM micrographs of an adhered parallelepiped at the SiO_2_ NW interface after (a, b) 15 min and (c) 30 min of immersion in HF buffer. AFM imaging of a pyramid embedded in the SiO_2_ NWs, etched by a bath of (d) 5 min, (e) 20 min, and (f) 30 min.

The roughness, ascribed to the presence of the embedded SiO_2_ NWs, decreases significantly due to the removal of the SiO_2_ NWs exposed to the etching solution. The residual roughness of the structure surface after the HF buffer bath (see Figure 6) is ascribed to the dispersion in the diameter of the SiO_2_ NWs emerging from the polymerized surface, determining different etching stages for each SiO_2_ NW. Therefore, a better control on the SiO_2_ NW diameter during the CVD process would lead to smoother surfaces in the cases where the SiO_2_ NWs emerge from the polymerized volume. These findings were supported by SEM and AFM observations. In Figure 6, we compare the etching process for both the kinds of structures (parallelepiped and pyramid), presenting details in the order of few hundreds of nanometers. Figure 6a shows the SEM image of a parallelepiped after 15 min in the HF buffer bath. The higher magnification image in Figure 6b shows some SiO_2_ NWs still emerging from the surface. However, after 30 min of etching, the surface of the parallelepiped is totally smooth, and SiO_2_ NWs are completely absent (Figure 6c), indicating that a bath lasting between 15 and 30 min is enough to remove the excess of SiO_2_ NWs but not to penetrate inside the resin and eliminate those embedded.

Figure 6d–f shows the AFM imaging of a pyramidal structure after the same etching intervals, where similar results are obtained. In particular, in Figure 6d, the pyramid, bathed in HF for 5 min, is visibly embedded in the NW layer. As a consequence, the AFM imaging reveals only the apex of the pyramid but not all the structure. A further bath of 15 min removes most of the SiO_2_ NWs (Figure 6e). After another etching of 10 min (Figure 6f), meaning a total etching of 30 min, the wires are completely removed from the pyramid surface, allowing us to reveal the full volume of the object (20 × 20 × 6 μm^3^). Hence, in both cases, an optimal etching in the range of 15–30 min is estimated to completely remove the exceeding SiO_2_ NWs. Figure 6 also allows evaluating the maximum achievable resolution, from the sharpness of the corners and edges of both samples, in particular for the apical part of the pyramid, which allows us to assess a sub-micrometric resolution in the same order of magnitude as that of the bare photoresist. In Figure 6f, the pyramid surface is completely free from the SiO_2_ NWs, revealing that they do not affect the TP-DLW resolution. In fact, the intertwining of the polymeric segments composing the 3D object are perfectly recognizable both in the top and bottom layers, showing that the diameter of the strips is around 400 nm everywhere. This result demonstrates that by matching the refractive indices of the SiO_2_ NWs and the photoresist, the resolution of the TP-DLW technology is practically preserved.

Moreover, we observe that in the case of small structures, the total 30 min of etching generated few nanoholes, whose diameter is 50–80 nm, due to the removal of the superficial SiO_2_ NWs, embedded in the polymer and crossing the pyramid surface. This effect can be controlled and limited by finely tuning the etching time. However, an increase in the surface roughness is plausible because of the dispersion in the NW diameter, as the larger the diameter the longer the correct etching time. We conclude that this effect could be fully avoided if monodisperse diameter NWs were available.

**Figure 7:**
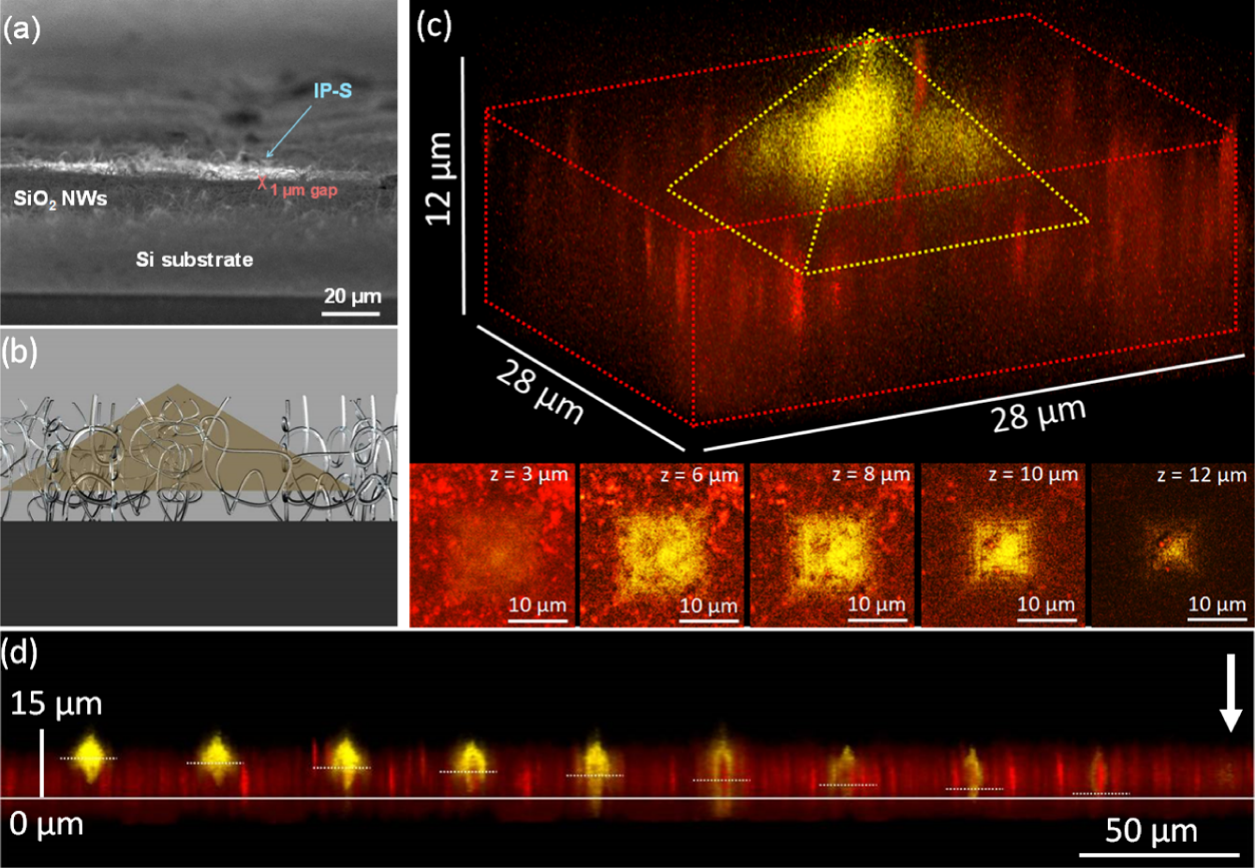
(a) SEM image of the cross section of an FRPN showing the gap between the Si substrate and the sample printed with the IP-S resin. (b) Cartoon depicting (a) for a clearer interpretation and (c) 3D CFM reconstruction of a pyramid created in the SiO_2_ NW network, with a sacrificial layer. The CFM images, taken at different focal planes, reveal the sacrificial layer, the base of the pyramid embedded in the NWs, and the emerging tip. (d) Cross section reconstructed from a stack of images of a series of pyramids printed with a decreasing gap between the structure bottom and the substrate from 9 to 0 μm. The SiO_2_ NW layer was infiltrated with a dye (E120) with a fluorescence peak at 630 nm (red), well different from the 540 nm of the used resist (yellow). The solid and the dashed white line trace the Si substrate plane and the structure base, respectively. The position of the pyramid with zero gap, poorly visible, is marked with the white arrow.

In the second configuration analyzed, samples were printed, leaving a gap between the bottom of the structure and the Si substrate (see Figures 3b and 7). In this case, an etching time in the range of 10–30 min was enough for the HF buffer to selectively dissolve the SiO_2_ NWs within the gaps, leaving the created objects completely free. Thus, the SiO_2_ NWs act here as a sacrificial layer that can be removed on demand. It must be noted that the necessary etching time shortens when the gap clearance increases, so the objects printed at larger gaps are detached first. Hence, the etching time to eliminate the sacrificial layer can be tuned by controlling the clearance of the gap and therefore the diffusion kinetics of the HF. Even in this case, both a larger (500 × 500 × 2 μm^3^) parallelepiped and arrays of small (20 × 20 × 6 μm^3^) pyramids could be printed. The parallelepiped was fabricated according to the scheme in Figure 3b and analyzed by SEM imaging (Figure 7a), which reveals the gap between the FPRN structure and the substrate surface. The scheme of the pyramid fabrication is shown in Figure 7b; after printing, the objects were analyzed by confocal fluorescence microscopy (CFM), as shown in Figure 7c,d. Here, the SiO_2_ NWs have been dyed with E120, a food coloring which features a fluorescence peak at 630 nm, well-distinct from that of the IP-S resin (540 nm), allowing us to reveal the gap between the substrate and the pyramid base. Figure 7c shows a pyramid presenting a gap of 6 μm. The sample was scanned at different focal planes, as shown at the bottom of the figure. The CFM imaging of the sample at different *z*-values clearly shows that while the pyramid tip emerges from the SiO_2_ NW layer, the base is trapped inside. Moreover, at a lower *z*-value, in the image at *z* = 3 μm, we did not detect any fluorescence from the IP-S pyramid but only from the dyed NWs, thus demonstrating the gap. By summing the contributions of the pictures taken at the different focal planes, the 3D CFM profile of the pyramid and the sacrificial layer was reconstructed, as shown in the top of Figure 7c.

**Figure 8:**
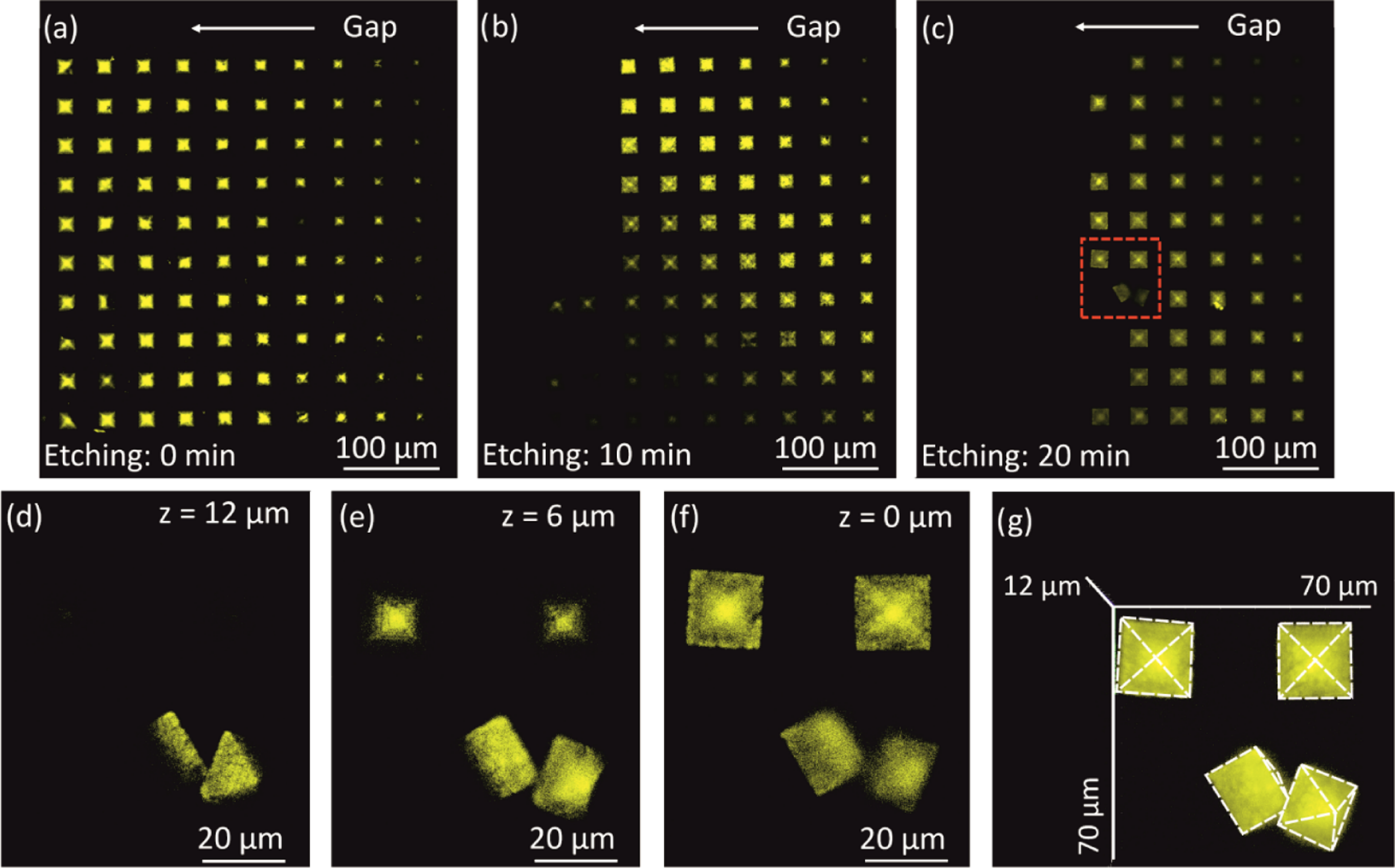
CFM images of a 10 × 10 array of FRPN pyramids, where all the elements in each column have the same gap clearance from 0 (right) to 9 μm (left). (a) Before etching and (b) after etching for 10 min and (c) for 20 min. (d–g) Magnification of the detail of (b), taken at (d) *z* = 12 μm, (e) *z* = 6 μm, and (f) *z* = 12 μm, (g) 3D reconstruction. An upside-down pyramid is recognizable and two others that have been removed from the substrate appear in a rotated position.

To demonstrate the possibility to selectively control the structure removal, an array of 10 × 10 pyramids with an increasing gap from 0 (the column on the right) to 9 μm was printed, as Figure 7d shows. Figure 8a shows the structure before the HF buffer etching. The different *z* quotes of each column in the matrix are revealed by the *x*–*y* footprint of the fluorescence image, smaller for the deeper objects because of their pyramidal shape. Figure 8b shows the same matrix after immersion in the HF buffer for 10 min. All the structures with a gap below 7 μm are still attached to the fabrication substrate, and the structures with a 9 μm gap are completely removed, and only six structures with a 7–8 μm gap are still present on the substrate. A further HF bath of 10 min (Figure 8c) causes the detachment of the pyramids with a gap of >5 μm. Some pyramids with a gap of 5 μm, in the red square, have been removed but are still present on the substrate. A magnification of these structures is shown in Figure 8d–g, where the CFM imaging at different planes and the 3D reconstruction clearly demonstrate that these pyramids are not trapped in the NWs anymore. The buffer was not stirred during the etching process given the delicacy of the structures; therefore, the small irregularities observed in the diagonals of the pyramids matrix are likely to be caused by possible gradients of the fluoride ion concentration in the bath. In any case, the possibility to tuning the etching time between the gap and the exposed surface adds a further degree of freedom in the fabrication process in the case of multistep fabrications.

**Figure 9:**
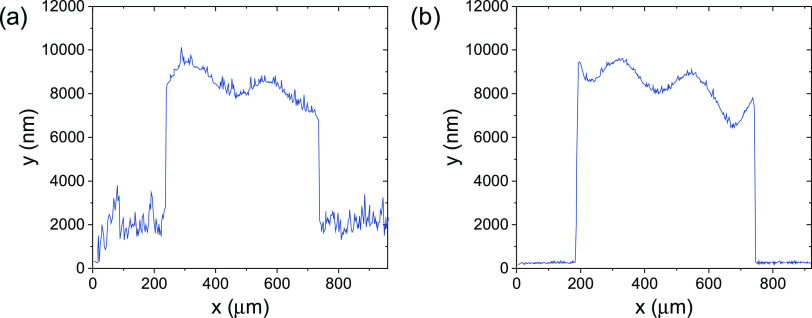
Profilometer measurements of an adhered (500 × 500 × 8 μm^3^) parallelepiped at the SiO_2_ NW interface following the shell and scaffold approach (a) before and (b) after 15 min of immersion in HF buffer. The decrease in the surface roughness indicates the successful etching of the exposed SiO_2_ NWs.

Finally, hollow structures were also successfully manufactured according to the shell and scaffold printing strategy, often used in the additive manufacturing domain to considerably shorten the processing time when larger solid objects have to be fabricated. In this standard fabrication method, only an external shell, together with an internal structure that prevents the collapse of the object, is polymerized by TP-DLW. After a standard development stage in PGMEA + IPA, the unpolymerized resist remaining inside of the shell is cured by single-photon absorption exposing the sample to a UV (360 nm) light source.^36,37^ In our case, the object is built as a hollow structure (shell) with the first layer printed at the interface with the Si substrate. The internal structure being printed here is made redundant by the presence of the SiO_2_ NWs acting as the scaffold in preventing the collapse of the thin shell, considerably shortening the process time. Figure 9 shows the profilometry of a (500 × 500 × 8 μm^3^) parallelepiped before and after the etching with the HF buffer. As in the case of the solid objects, the high roughness associated with the SiO_2_ NWs is strongly decreased after 15 min of etching. In this approach, the aim is to etch only the excess of SiO_2_ NWs out of the shell, so the time is expected to be the same as for solid objects (Figure 3a). Profilometry results evidence that the SiO_2_ NWs are able to provide an internal scaffold with a self-standing structure and prove that the SiO_2_ NWs inside the shell remain unaltered. Hence, this strategy can be adopted in TP-DLW when large volumes are needed, reducing significantly the printing times.^38,39^

Finally, it is important to point out that the ability to print structures attached to the SiO_2_ NWs eliminates the need to be exactly at the interface with the substrate for the proper anchoring of the object to be manufactured. This would eliminate the necessity of using the complex autofocus system for the interface detection in the DLW devices. Instead, our approach makes possible to have an effective range of *z* heights, determined by the thickness of the nanowires layer, from which the manufacture could start, making the autofocus system redundant.

### 3.2 Mechanical Characterization of the FRPN

**Figure 10:**
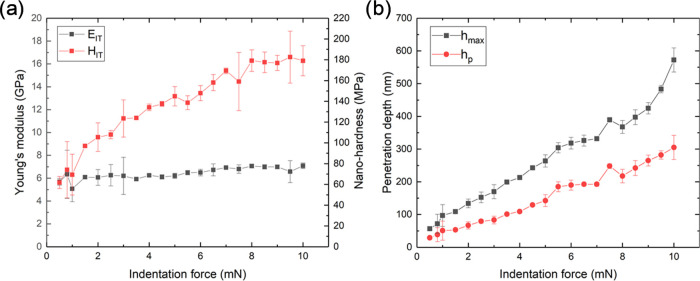
(a) Young’s modulus (*E*_IT_) and nanohardness (*H*_IT_) as a function of the indentation load; (b) Maximum indentation depth (*h*_max_) and residual depth (*h*_p_) as a function of the indentation force. For both plots, each datapoint is the average of five different indentations and is reported in the corresponding scatter band.

The mechanical properties of the manufactured PFPN were measured by nanoindentation, on the (500 × 500 × 8 μm^3^) parallelepiped, to have the thicker possible nanocomposite layer. Figure 10 reports the results of the nanomechanical characterization, as obtained from varying the indentation load *P*_max_ between 0.5 and 10 mN. Each datapoint in the figure represents the average value with its associated scatter band corresponding to the data obtained from five different indentations. In particular, Figure 10a reports the evolution of Young’s modulus (*E*_IT_) and of the nanohardness (*H*_IT_) as a function of the indentation load. Results revealed a significant increase in *H*_IT_ with the indentation force up to a value of 8 mN. Above this value, it reaches a plateau for higher indentation loads. On the other hand, the *E*_IT_ remains constant throughout the whole range of indentation loads studied (0.5–10 mN).

Nevertheless, as it can be seen in Figure 10b, the maximum penetration reached (*h*_max_) during the indentation is always below 700 nm, i.e., lower than 10% of the thickness of the nanocomposite. Therefore, it can be concluded that the studied region, either during plastic and elastic deformation, will not be affected by the presence of the Si substrate. More details about these experiments are in the Supporting Information.

**Figure 11:**
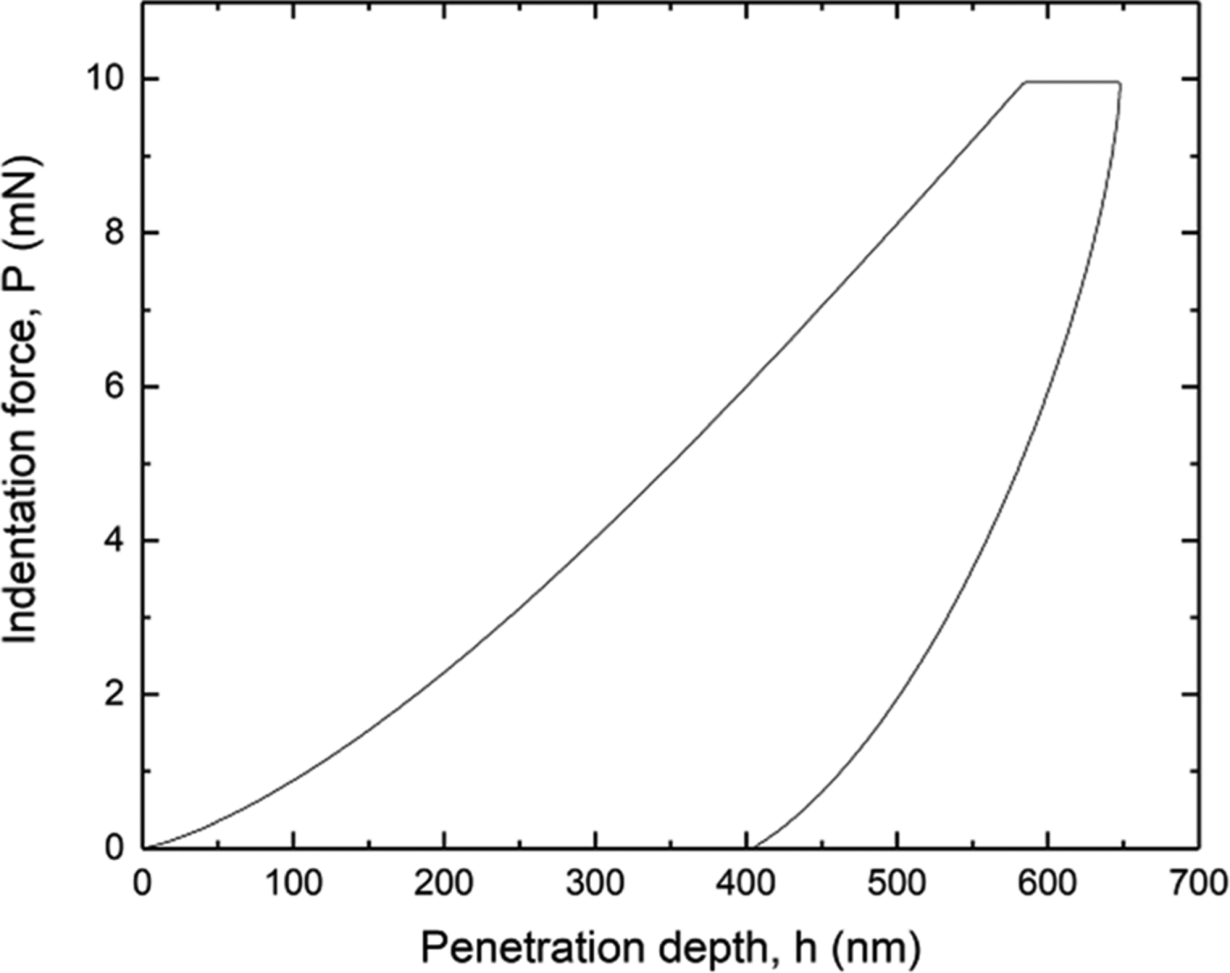
Indentation load vs penetration depth curve for the FRPN fabricated in this work.

According to these results, an indentation load equal to 10 mN was chosen to evaluate the mechanical properties at the nanoscale of the nanocomposite since under these conditions, both *E*_IT_ and *H*_IT_ are in the plateau region (i.e., maximum values that can be obtained). Then, a matrix of 10 × 8 indentation points was carried out with 100 μm spacing between each indentation. Hence, the indentation area is about 87 μm^2^ (see the Supporting Information), indicating that the analyzed zone is much larger than the typical size of the nanofiber reinforcements. As a consequence, indentation tests provide effective results of the FRPN layer averaged within the volume of the process zone, i.e., inhomogeneities in mechanical properties present on a lower length scale are not considered. An example of force versus indentation depth curve is shown in Figure 11.

The indentation tests revealed that the FRPN mechanical properties are characterized by a value of Young’s modulus and nanohardness equal to 8.63 ± 0.78 GPa and 120.06 ± 10.77 MPa, respectively. With the purpose of evaluating how the use of SiO_2_ NW substrates allows enhancing the mechanical properties of the obtained material, nanoindentation experiments were carried out also on the bare polymeric matrix. The indentation tests showed a marked reduction of both Young’s modulus and nanohardness. In fact, while Young’s modulus of the polymeric matrix is equal to 1.94 ± 0.10 GPa, the nanohardness reaches a mean value of 33.47 ± 0.35 MPa, as given in Table 1. These results evidence that the FRPN exhibits significantly enhanced mechanical properties compared to the bare polymer, with an increase of approximately 4 times in Young’s modulus and nanohardness values. To the best of our knowledge, this is the first work where the actual mechanical properties of an FRPN prepared by TP-DLW are measured. Other authors recently reported on the mechanical properties of zeolite-based nanocomposites, also prepared by TP-DLW. In their case, they observed an increase in the shear modulus of also approximately 4 times when the zeolite filler was at 70%.^40^ However, the values obtained were in the range of 10–100 MPa, significantly below ours. Therefore, these materials are promising for structural applications in the nanotechnology sector that require higher structural yields, such as in microfluidics.

**Table 1:** Comparison of Young’s modulus and nanohardness values for bare polymeric resin (IP-S) and the FRPN loaded with SiO_2_ NWs printed via TP-DLW in this work

	Young’s modulus (GPa)	Nanohardness (MPa)
IP-S	1.94 ± 0.10	33.47 ± 0.35
FRPN	8.63 ± 0.78	10.77

## 4 Conclusions

The use of transparent fillers as SiO_2_ NWs together with photosensitive resins allows for the immediate implementation of TP-DLW in the fabrication of FRPN 3D objects, as long as the refractive index of the glassy filler and the polymeric matrix match. Even if these indices do not match perfectly, in this study, we have proven that different objects can be successfully manufactured through an SiO_2_ NW layer using a commercial resist (IP-S). This allowed obtaining an FRPN with high filling volume fractions, up to 70%. Under optimal conditions (i.e., with a more accurate refractive index matching), this percentage could be raised up to saturation, being able to manufacture 3D glassy objects with sub-micrometric resolution, in analogy with the results recently reported by Kotz et al.^18^ Moreover, the use of substrates containing SiO_2_ NWs offers many practical advantages in the TP-DLW technique as it allows for the fabrication of high-resolution structures with improved mechanical features. This was demonstrated by printing objects at either the interface with the Si wafer or leaving a variable gap. In the first case, the structure is permanently attached to the substrate. In the second case, a sacrificial layer that can be removed under well-controlled conditions is created. This allows releasing the fabricated object on demand. Moreover, the SiO_2_ NWs can provide a robust internal scaffold when hollow structures are manufactured using the shell and scaffold printing strategy, shortening considerably the fabrication time. Finally, the use of such substrates makes a sophisticated autofocus system unnecessary.

On the other hand, the embedding of SiO_2_ NWs in a polymeric matrix for TP-DLW expands the range of materials with enhanced mechanical properties for TP-DLW. In fact, the mechanical characterization at the nanoscale shows a remarkable improvement in the nanohardness and elastic modulus of the FRPN, around 4 times higher compared to that the bare IP-S resist. This makes the proposed technique well-suited for applications requiring higher mechanical performance, as in advanced microfluidics or micromechanics.

## Supporting Information Available

Detailed explanation of the mechanical characterization of the FRPN (PDF)
