# Supplementary material for: High-Resolution 3D Fabrication of Glass Fiber-Reinforced Polymer Nanocomposite (FRPN) Objects by Two-Photon Direct Laser Writing
Source: ACS Appl Mater Interfaces. 2022 Apr 8;14(15):17754–62. doi: 10.1021/acsami.1c21708 (PMC9026244; doi:10.1021/acsami.1c21708)
Supplement: Supplementary file 1 — am1c21708_si_001.pdf [file am1c21708_si_001.pdf]

SUPPORTING INFORMATION

High Resolution 3D Fabrication of Glass Fiber-Reinforced Polymer Nanocomposite (FRPN) Objects by Two-Photon Direct Laser Writing

Tiziana Ritacco[#], Wera Di Cianni[,#], Dario Perziano, Pietro Magarò, Annalisa Convertino, Carmine Maletta, Antonio De Luca, Alberto Sanz de León[*], Michele Giocondo*

Tiziana Ritacco, Wera Di Cianni, Antonio De Luca, Michele Giocondo
Institute of Nanotechnology – Nanotec Consiglio Nazionale delle Ricerche - Sede di Cosenza. Ponte P. Bucci - Cubo 33C, Rende 87036, Italy
E-mail: weradicianni@gmail.com

Tiziana Ritacco, Wera Di Cianni, Dario Perziano, Antonio De Luca
University of Calabria Physics Department, 87036 Arcavacata di Rende (CS), Italy

Annalisa Convertino
Institute for Microelectronics and Microsystems – IMM Consiglio Nazionale delle Ricerche, via del Fosso del Cavaliere 100, 00133 Roma, Italy

Pietro Magarò, Carmine Maletta
University of Calabria, Department of Mechanical, Energy and Management Engineering, Cubo 44C, Arcavacata di Rende, 87036, Italy

Wera Di Cianni, Alberto Sanz de León
Departamento de Ciencia de los Materiales, I. M. y Q. I., IMEYMAT, Facultad de Ciencias, Universidad de Cádiz, Campus Río San Pedro, s/n, 11510 Puerto Real (Cádiz), Spain
E-mail: alberto.sanzdeleon@uca.es

[#]These authors contributed equally to this work.

A typical force-displacement curve obtained from instrumented indentation is illustrated in **Figure S1**. The indentation curve ($P - h$), in fact, can be used to calculate the nano-hardness and Young's modulus.

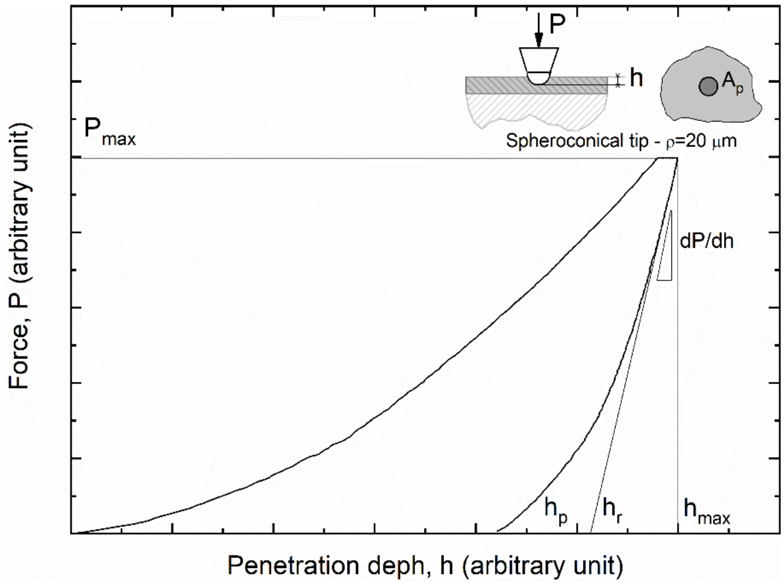

Figure S1. Typical nano indentation curve force (P) vs indentation depth (h) together with significant indentation parameters used to calculate the indentation nano harness (H_{IT}) and Young's modulus (E_{IT})

The nano-hardness (H_{IT}) can be directly measured from the indentation curve as reported in the following equation:

$$H_{IT} = \frac{P_{max}}{A_p(h_p)} \quad (1)$$

where A_p, about 87 μm^2, is the projected area after indentation, that is obtained geometrically as a function of the residual depth h_p. The reduced Young's modulus (E_r) can be calculated from the contact stiffness ($S = {dP}/{dh}$), that is the slope of the initial path of the unloading curve (see **Figure S1**), according to the standard ISO 14577-1 (2002)[1], based on the Oliver and Pharr (1992) method[2]:

$$E_r = \frac{1}{2}\sqrt{\frac{\pi}{A_p(h_p)}}S \quad (2)$$

Where ${dP}/{dh}$ is calculated in the range 0.4-0.98 P_{max}. The indentation modulus of the material, E_{IT}, can be obtained from E_r by considering the elastic properties of the indenter tip (E_i=1140 GPa and v_i=0.07 for diamond):

$$E_{IT} = (1 - v^2)\left(\frac{1}{E_r} - \frac{1 - v_i^2}{E_i}\right)^{-1} \quad (3)$$

Due to the average thickness of the NWs layer over the fabrication substrate, that is about 8 μm., a proper indentation load should be chosen in order to ensure that the plastic and elastic volume, involved during the test, is not affected by edge effects. With this purpose, an indentation matrix was performed on the sample in which the indentation load was varied in the range 0.5 mN-10 mN. This was made to avoid possible estimation errors of both Young's modulus and material hardness due to load/size effects as well as to keep the maximum indentation depth (h_{max}) below 10% of the composite thickness.

(1) ISO 14577-1:2002. ISO 14577-1:2002. Metallic Materials – Instrumented Indentation Test for Hardness and Materials Parameters – Part 1: Test Method. Met. Mater. – instrumented indentation test hardness Mater. parameters – Part 1 test method. 2002.

(2) Oliver, W. C.; Pharr, G. M. An Improved Technique for Determining Hardness and Elastic Modulus Using Load and Displacement Sensing Indentation Experiments. J. Mater. Res. 1992, 7, 6, 1564–1583. https://doi.org/10.1557/jmr.1992.1564.
